# Holographic Fabrication of Designed Functional Defect Lines in Photonic Crystal Lattice Using a Spatial Light Modulator

**DOI:** 10.3390/mi7040059

**Published:** 2016-04-01

**Authors:** Jeffrey Lutkenhaus, David Lowell, David George, Hualiang Zhang, Yuankun Lin

**Affiliations:** 1 Department of Physics, University of North Texas, Denton, TX 76203, USA; jeff.lutkenhaus@gmail.com (J.L.); davidlowell@my.unt.edu (D.L.); davidgeorge2@my.unt.edu (D.G.); 2 ECE Department, University of Massachusetts Lowell, Lowell, MA 01854, USA; hualiang_zhang@uml.edu; 3 Department of Electrical Engineering, University of North Texas, Denton, TX 76203, USA

**Keywords:** microstructure fabrication, photonic crystals, spatial light modulators, holographic lithography

## Abstract

We report the holographic fabrication of designed defect lines in photonic crystal lattices through phase engineering using a spatial light modulator (SLM). The diffracted beams from the SLM not only carry the defect’s content but also the defect related phase-shifting information. The phase-shifting induced lattice shifting in photonic lattices around the defects in three-beam interference is less than the one produced by five-beam interference due to the alternating shifting in lattice in three beam interference. By designing the defect line at a 45 degree orientation and using three-beam interference, the defect orientation can be aligned with the background photonic lattice, and the shifting is only in one side of the defect line, in agreement with the theory. Finally, a new design for the integration of functional defect lines in a background phase pattern reduces the relative phase shift of the defect and utilizes the different diffraction efficiency between the defect line and background phase pattern. We demonstrate that the desired and functional defect lattice can be registered into the background lattice through the direct imaging of designed phase patterns.

## 1. Introduction

Photonic crystals (PhCs) are nano/micro photonic structures in which the dielectric constant is periodically modulated on a length scale comparable to the desired operation wavelength [1]. PhCs have been the focus of intense research for the many potential applications on the manipulation and control of light, including one-dimension PhC as fiber Bragg gratings for optical switches and laser cavities [2], 3D PhC cavity lasers with high quality factor [3], PhC waveguides for integrated circuits or miniaturization of photonic devices [4], and cloaking [5].

In PhCs, photons with a wavelength within the PhC bandgap are forbidden to propagate in the PhC [1]. If defects are added to the PhC, photons with a wavelength in the photonic bandgap will be allowed along the defect [1,3,4,6]. Engineered defects in PhCs not only fundamentally change their physical properties, but also offer new possibilities for designing devices with new functionalities [6]. Carefully designed point-like defects in PhC can be used as compact laser nanocavities, efficient devices to couple light into PhC-based waveguides, or resonant add-drop filters [6]. In addition, linear defects in PhCs can act as efficient waveguides for future integrated optical circuits [4,7].

Conventional holographic lithography uses bulk optical elements to control the number of interfering beams, interference angles, polarization, and phase of the interfering beams, for the simultaneous patterning of PhCs template in photoresist [8,9,10,11,12,13,14,15,16,17]. The use of single-optical elements, including phase masks [10,11,12,13,14], prisms [15], and reflective optical elements [17,18], has significantly reduced the complexity of the optical setup for laser holographic lithography. Holographic lithography has also been applied to fabrication of PhC templates and desired defects simultaneously [19,20,21,22,23].

Recently, spatial light modulators (SLMs) have been used to engineer the phases of interfering beams through computer generated holograms to fabricate various photonic structures, such as quasi-periodic [24,25], periodic [25,26,27], multi-periodic [28], and chiral photonic structures [29]. Several methods have been used to incorporate desired functional defects into the PhC lattice through SLM-based holographic lithography [23,30,31,32,33,34,35,36,37,38,39,40,41]. Pioneering works using SLM as a masks or an adaptive optical element have demonstrated the formation of complex point and line defects and zigzag waveguides in PhC [23,41]. Desired point defects can be fabricated into background PhC lattices using an SLM by superposing a Bessel beam into the background wave-field [30,31]. By specifying the phases [32] or gray levels of simple geometric phase patterns [33] and displaying them on the SLM, point or line defects can be embedded in PhCs. Gradient photonic lattices can be generated through spatially varying pixel-by-pixel phase engineering of phase patterns [34], or through production of hexagonal lattice wave fields with a gradient basis [35]. Spatially variant photonic crystal lattices have been fabricated with the SLM using an innovative synthesis approach that calculates the structure of the lattice while varying lattice orientation, lattice spacing, and filling fraction [36,37,38,39,40]. Although these methods were successful for the fabrication of functional PhCs using an SLM, arbitrary designs of phase patterns can result in an assignment of several gray levels on a single pixel, thus having a super-lattice effect.

In this paper, we intend to realize direct imaging of functional defect lines in PhCs through holographic lithography by assigning one gray level for each pixel in the SLM. Because we integrate the functional defects in the phase pattern, the interfering beams not only carry the defect’s content but also the defect related phase information. The phase shift of interfering beams near the functional defects will modify the photonic lattice near the defects. After we fully understand the phase-related lattice shift and compare them in five-beam interference patterns with those in three-beam interference patterns (Section 3, Section 4 and Section 5), we design and realize functional defects that can be registered in the PhC lattices in Section 6.

## 2. Experimental Setup and Theory

In the experimental setup, an SLM is used for the generation of multiple-beam interference and control of the phase of interfering beams [27,33]. Because we intend to understand and gain knowledge of direct imaging by SLM, we did not use a microscope objective lens. A 532 nm laser beam (Cobolt Samba 50 mW) was expanded, collimated, and incident onto a phase-only SLM (Holoeye PLUTO) at an incident angle of 4 degrees. The laser was modulated by the phase pattern displayed on the SLM and imaged by a 4f (*f*_1_ = 400 mm and *f*_2_ = 200 mm) imaging system. A Fourier filter was placed at the Fourier plane of the *f*_1_ lens in order to block the undesired beams and allow the desired ones. A sample holder was mounted on a 3D translation stage to record the interference pattern in a photoresist mixture. The photoresist mixture was prepared by mixing the following components: dipentaerythritol hexa-/penta-acrylate (DPHPA) monomer, *N*-vinyl pyrrolidinone chain extender, *N*-phenyl glycine co-initiator, photoinitiator rose bengal, and triethylamine. The mixture was spin-coated onto a glass slide at 3000 rpm for 30 s. After exposure to the interference pattern, the sample was developed in propylene glycol methyl ether acetate for 3–5 min, rinsed in isopropanol for 1 min, and air dried.

**Figure 1:**
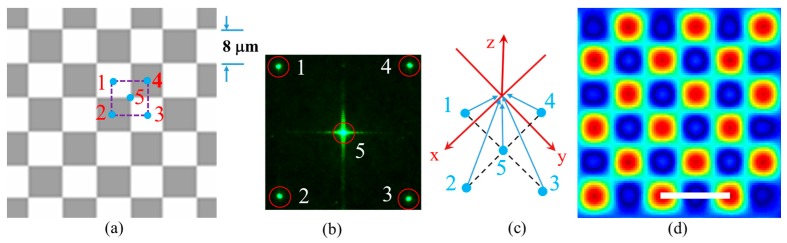
(**a**) An enlarged view of the checkerboard phase pattern displayed on the SLM. The dashed purple square indicates the size of one unit cell of 8 × 8 μm^2^. (**b**) CCD image of the diffraction pattern at the Fourier plane. The red circles indicate beams passing through the Fourier filter. (**c**) Scheme of the five beam interference arranged four-fold symmetrically. Beam 5 propagates along the *z*-axis. (**d**) Simulated intensity distribution for the interference of the four first order beams with the central zero order beam after the 4f imaging system. Scale bar is for 8 μm.

A square checkerboard phase pattern was displayed on the SLM and used to generate multiple diffracted beams. Figure 1a shows an enlarged view of the checkerboard phase pattern; it is comprised of 6 × 6 unit cells, as an example. The dashed purple square indicates one of the unit cells with the direction of periodicity at 45 degrees with respect to horizontal and vertical directions. The size of the unit cell is 8 × 8 μm^2^, which is also the size of one pixel in the SLM (the SLM has entire 1920 × 1080 pixel area). The gray level of each pixel can be individually set to desired values. The phase pattern displayed on the SLM generated four first order diffracted beams, labeled 1, 2, 3, and 4 in Figure 1a, and one central zero order beam, labeled 5 in Figure 1a, when the laser was incident onto the SLM. The phases of the diffracted beams (1, 2, 3, and 4) are determined by the individual gray level of the numbered pixels in Figure 1a, using the formula ϕ=(2π/255)×(gray level/4) [34]. The factor of 4 in the formula is due to the fact that a quarter of each pixel is included in the dashed purple unit cell in Figure 1a. Figure 1b shows an example of the diffraction pattern of the laser beams diffracted by the checkerboard phase pattern on the SLM at the Fourier plane of lens *f*_1_. The pattern was recorded by a charge-coupled device (CCD) camera. Surrounding the 0th order diffraction in the center, four 1st order diffractions (labeled 1, 2, 3, and 4) form a pattern that has 4-fold symmetry. A Fourier filter is used to let the beams inside the red circles pass through. Propagating throughlens *f*_2_, these five beams are overlapped as arranged in a coordinate system as shown in Figure 1c. The five beams are represented by the following equations:

(1) $$E_{1}\left(r,t\right)=E_{1}\text{cos}\left[\left(k\text{ sin}\theta \right)y+\left(k\text{ cos}\theta \right)z-\omega t+\phi_{1}\right]$$

(2) $$E_{2}\left(r,t\right)=E_{2}\text{cos}\left[\left(-k\text{ sin}\theta \right)\;x+\left(k\text{ cos}\theta \right)z-\omega t+\phi_{2}\right]$$

(3) $$E_{3}\left(r,t\right)=E_{3}\text{cos}\left[\left(-k\text{ sin}\theta \right)y+\left(k\text{ cos}\theta \right)z-\omega t+\phi_{3}\right]$$

(4) $$E_{4}\left(r,t\right)=E_{4}\text{cos}\left[\left(k\text{ sin}\theta \right)x+\left(k\text{ cos}\theta \right)z-\omega t+\phi_{4}\right]$$

(5) $$E_{5}\left(r,t\right)=E_{5}\text{cos}\left[kz-\omega t+\phi_{5}\right]$$

where *E* is the electric field, *k* is the wave vector, and ϕ is the initial phase of the beam. An interference pattern is formed when the five beams are overlapped. The intensity distribution in the interference pattern can be determined by the following equation and the five-beam interference pattern in *x*-*y* plane is shown in Figure 1d:

(6) $$I\left(r\right)=I_{0}+\Delta I\left(r\right)=\langle \overset{5}{\underset{i=1}{\sum }}E_{i}^{2}\left(r,t\right)\rangle +\overset{5}{\underset{i<j}{\sum }}E_{i}\cdot E_{j}\text{cos}\left[\left(k_{j}-k_{i}\right)\cdot r+\left(\phi_{j}-\phi_{i}\right)\right]$$

If we integrate the functional defects in the phase pattern, the first order diffracted beam carries the spatial frequency information of the defect line. The amplitude patterns associated with each first-order diffracted beam are shifted with the same spatial frequency from the origin by an amount of sinθ/λ [33]. When the diffracted beams are overlapped for the interference, the interfering beams not only carry the defect’s content but also the defect related phase information. The phase shift of interfering beams near the functional defects will modify the photonic lattice near the defects.

## 3. Direct Imaging of Functional Line Defects in Square PhC Lattice through Five-Beam Interference

**Figure 2:**
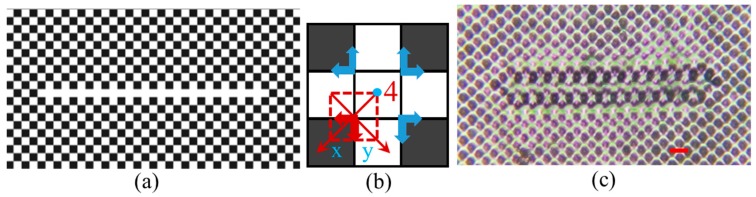
(**a**) Checkerboard phase pattern (with gray levels 30 and 255) and designed line defect (gray level 255). Each bright or dark square in the pattern represents a single square SLM pixel. (**b**) Close-up of portion of line defect in phase pattern. Arrows indicate the direction of the lattice shift due to the phase shift. Red square represents the unit cell for calculating phases of the interfering beams. (**c**) Optical microscope image of fabricated structures in DPHPA. Scale bar is for 8 μm.

In this section, we study the lattice shift during the direct imaging of functional defect lines in the PhC lattice through five-beam interference lithography. The checkerboard pattern is used as a background phase pattern with a bright gray level of 255 and a dark gray level of 30 as shown in Figure 2a. For the functional defect line, the gray levels of a line of pixels are changed to 255 to create a white line defect in the background phase as shown in Figure 2a. In Figure 2b, an enlarged view of the line defect in the phase pattern is shown. The red dashed square indicates a unit cell from which the phase of the interfering beams can be calculated. Before adding the defect line to the phase pattern, the pixel for beam 4 in the defect phase shown in Figure 2b had a gray level of 30. After adding the defect, the gray level of the pixel for beam 4 is changed to 255. Thus, the phase of beam 4 in the defect line in Figure 2b is shifted by Δ × 2π, where Δ = ((255–30)/255) × 0.25. The lattice shift in the defect region of the five-beam interference pattern due to beam 4 can be written as:

(7) $$\begin{array}{ll} \Delta I\left(\text{related to beam }4\right)= & E_{1}\cdot E_{4}\text{cos}\left[\left(-k\;\text{sin}\theta \right)x+\left(k\;\text{sin}\theta \right)y+\left(\phi_{1}-\phi_{4}\right)-\Delta \cdot 2\pi \right]\\ & +E_{2}\cdot E_{4}\text{cos}\left[\left(-2k\;\text{sin}\theta \right)x+\left(\phi_{2}-\delta_{4}\right)-\Delta \cdot 2\pi \right]\\ & +E_{3}\cdot E_{4}\text{cos}\left[\left(-k\;\text{sin}\theta \right)x-\left(k\;\text{sin}\theta \right)y+\left(\phi_{3}-\phi_{4}\right)-\Delta \cdot 2\pi \right]\\ & +E_{4}\cdot E_{5}\text{cos}\left[\left(k\;\text{sin}\theta \right)x+\left(k\;\text{cos}\theta -k\right)z+\left(\phi_{4}-\phi_{5}\right)+\Delta \cdot 2\pi \right] \end{array}$$

We define the lattice constant in the *x*-*y* plane to be a=λ/sinθ and the lattice constant in the *z*-direction to be c=λ/(1−cosθ), where λ is the wavelength of the interfering beam. Based on these lattice constants, Equation (7) can be rewritten as:

(8) $$\begin{array}{ll} \Delta I\;\left(\text{related to beam }4\right)= & E_{1}\cdot E_{4}\text{cos}\left[\left(-2\pi /a\right)x+\left(2\pi /a\right)y+\left(\phi_{1}-\phi_{4}\right)-\Delta \cdot 2\pi \right]\;\\ & +E_{2}\cdot E_{4}\text{cos}\left[\left(-4\pi /a\right)x+\left(\phi_{2}-\phi_{4}\right)-\Delta \cdot 2\pi \right]\;\\ & +E_{3}\cdot E_{4}\text{cos}\left[\left(-2\pi /a\right)x-\left(2\pi /a\right)y+\left(\phi_{3}-\phi_{4}\right)-\Delta \cdot 2\pi \right]\;\\ & +E_{4}\cdot E_{5}\text{cos}\left[\left(2\pi /a\right)x+\left(2\pi /c\right)z+\left(\phi_{4}-\phi_{5}\right)+\Delta \cdot 2\pi \right]\; \end{array}$$

The lattice shift in the defect lattice relative to the background lattice due to the phase shift of Δ × 2π in beam 4 can be determined by the following Equation (9) (which is modified from Equation (8)):

(9) $$\begin{array}{ll} \Delta I\;\left(\text{beam }4\right)= & E_{1}\cdot E_{4}\text{cos}\left[2\pi \left(\left(-1/a\right)i+\left(1/a\right)j\right)\left(x+y+z+\Delta \left(0.5a-0.5a\pm 0.5c\right)\right)+\left(\phi_{1}-\phi_{4}\right)\right]\\ & +E_{2}\cdot E_{4}\text{cos}\left[2\pi \left(\left(-2/a\right)i\right)\left(x+y+z+\Delta \left(0.5a\pm 0.5a\pm 0.5c\right)\right)+\left(\phi_{2}-\phi_{4}\right)\right]\\ & +E_{3}\cdot E_{4}\text{cos}\left[2\pi \left(\left(-1/a\right)i+\left(-1/a\right)j\right)\left(x+y+z+\Delta \left(0.5a+0.5a\pm 0.5c\right)\right)+\left(\phi_{3}-\phi_{4}\right)\right]\\ & +E_{4}\cdot E_{5}\text{cos}\left[2\pi \left(\left(1/a\right)i+\left(-1/c\right)k\right)\left(x+y+z+\Delta \left(0.5a\pm 0.5a-0.5c\right)\right)+\left(\phi_{4}-\phi_{5}\right)\right]\; \end{array}$$

The *x* and *y* axes are labeled in Figure 2b. A phase shift of Δ × 2π results in a pattern shift of Δ(0.5a±0.5a±0.5c) in the defect region, indicated by the red arrows in Figure 2b. By applying different *x*- and *y*-axes, the same analysis can be applied to the unit cells immediately above, opposite to, or to the right of the dashed red square in Figure 2b. The blue arrows indicate the lattice shift directions for three unit cells. Thus, the theoretical analysis predicts that the photonic pattern near the defect line is stretched and squeezed periodically in the horizontal direction and shifted away vertically from the center of the defect line, leaving a gap on either side of the line defect. Figure 2c shows an optical microscope image of the fabricated PhC structures with designed defect line in DPHPA photoresist. Although a 3D pattern is predicted in Equation (6), a 2D PhC lattice is formed in Figure 2c when we use very thin film of DPHPA. In the region near the defect line, the lattice pattern is squeezed periodically in the horizontal direction and shifted away from the line center as predicted by the theory. 

**Figure 3:**
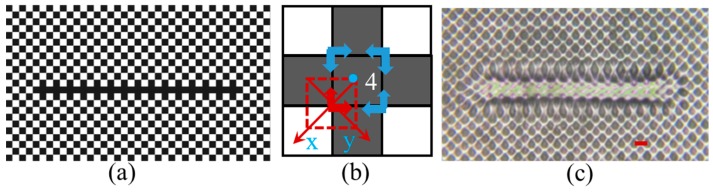
(**a**) Checkerboard phase pattern with black (gray level 30) defect line. (**b**) Enlarged view of line defect in phase pattern. Arrows indicate the direction of the lattice shift due to the presence of the line defect phase. The red square represents the unit cell for calculating phases of the beams. (**c**) Optical microscope image of line defect embedded in PhC lattice in DPHPA fabricated through holographic lithography. Scale bar is for 8 μm.

If the gray level for the pixels in the defect line is assigned to be 30 (in black) instead of 255 (in white), the phase shift of beam 4 as shown in Figure 3a is Δ = ((30−255)/255) × 0.25 relative to the background pattern. This causes an interference pattern shift in the direction of (−1, 1) or (−1, −1) for the 2D pattern in the defect region as shown by red arrows in Figure 3b, the opposite direction as shown by red arrows in Figure 2b. As indicated by the red and blue arrows in Figure 3b, the lattice in the defect region is expected to squeeze together and stretch away periodically in the horizontal direction and shifted toward the center of the defect line vertically. This is what has been seen in the interference pattern recorded in the DPHPA photoresist in Figure 3c. These studies prove that the diffracted beams not only carry the defect-content but also shifted-phase information. 

## 4. Direct Imaging of Functional Line Defects in Square PhC Lattice through Three-Beam Interference

**Figure 4:**
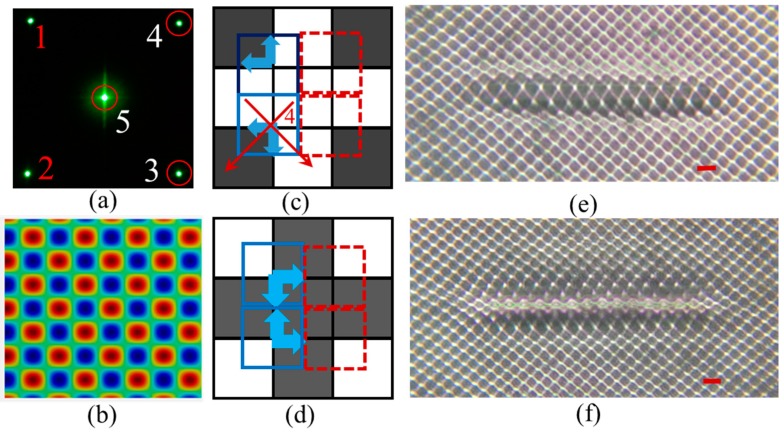
(**a**) CCD image of the diffraction pattern at the Fourier plane. The red circles indicate beams passing through the Fourier filter. (**b**) Interference pattern of two 1st order side beams with the central zero order beam. Scheme of white (gray level 255) (**c**) and dark (gray level of 33) (**d**) defect phase. Blue arrows indicate direction of the lattice shift due to the presence of the defect. (**e**,**f**) Optical microscope images of fabricated defects in PhC lattice using the defect phases in background phase pattern in (c,d), respectively. Scale bars are for 8 μm.

As shown in Figure 2b and Figure 3b, each unit cell carries the phase shift information of the diffracted beams and these phase effects cause unintended squeezing of the lattice near the defect line. In an attempt to mitigate the effects of the phase content in the diffracted beams, two of the beams (1 and 2 in red) were blocked at the Fourier filter as shown in Figure 4a. The two remaining diffracted beams (3 and 4) plus the central zero order beam (5) form an interference pattern, as shown in Figure 4b. The interference pattern still maintains a square lattice. Figure 4c,d are enlarged views the of phase patterns in Figure 2a and Figure 3a, respectively. The unit cell in the top dashed red square in Figure 4c,d has a shifted-phase contribution in diffracted beam 2, while the unit cell at the bottom dashed square contributes to the phase of beam 1. Because the diffracted beams (1 and 2) are blocked, only diffracted beams from the blue solid squares in Figure 4c,d carry the phase shift signal and form the interference pattern with shift directions as indicated by the blue arrows. In theory, it can be understood by the formula for three-beam interference and assuming a phase shift of Δ × 2π for beam 4:

(10) $$\begin{array}{ll} I\left(r\right)=I_{0}+\Delta I\left(r\right)= & I_{0}+E_{3}\cdot E_{4}\text{cos }[2\pi \left(\left(-1/a\right)i+\left(-1/a\right)j\right)\left(x+y+z+\Delta \left(0.5a+0.5a\pm 0.5c\right)\right)+\left(\phi_{3}-\phi_{4}\right)]\\ & +E_{3}\cdot E_{5}\text{cos}\left[2\pi \left(\left(-1/a\right)j+\left(-1/c\right)k\right)\left(x+y+z\right)+\left(\phi_{3}-\phi_{5}\right)\right]\;\\ & \;+E_{4}\cdot E_{5}\text{cos}\left[2\pi \left(\left(1/a\right)i+\left(-1/c\right)k\right)\left(x+y+z+\Delta \left(0.5a\pm 0.5a-0.5c\right)\right)+\left(\phi_{4}-\phi_{5}\right)\right] \end{array}$$

We fabricated PhC patterns with the designed defects by exposing the photoresist DPHPA to the interference pattern given by two first order diffracted beams (3 and 4) and the central beam 5. Figure 4e,f show optical microscope images of fabricated PhC patterns with designed defects in DPHPA using the designed defect lines in Figure 4c,d in the background phase pattern, respectively. The fabricated samples in Figure 4e,f show no squeezing or stretching in the horizontal direction, in contrast to the fabricated samples in Figure 2c and Figure 3c. In the vertical direction, the photonic lattice in Figure 4e is shifted away from the defect line center, similar to the phenomenon in Figure 2c, while the photonic lattice in Figure 4f is shifted toward the defect line center, similar to the phenomenon in Figure 3c. The theory predicts an alternating shifting in defect lattices in vertical direction because beams (1 and 2) are blocked by the Fourier filter. The width of the central squeezed region in vertical direction in Figure 4f is narrower than the one in Figure 3c due to the alternating shifting. From these results, there is an improvement in the direct imaging of functional defects, by using 2 + 1 interference rather than 4 + 1 interference.

## 5. 45-Degree Orientation of Functional Line Defect in Square PhC Lattice

**Figure 5:**
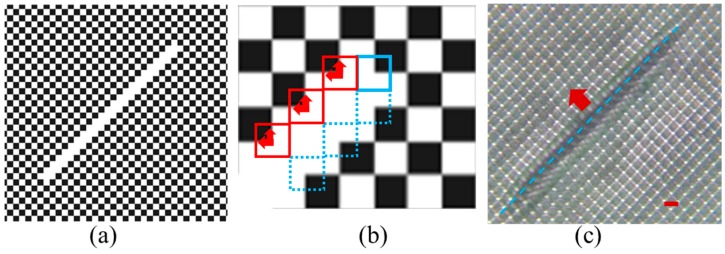
(**a**) Enlarged view of a diagonal line defect phase in the checkerboard phase pattern; (**b**) diagram of defect phase with accompanying lattice shifts indicated by the red arrows; (**c**) optical microscope image of fabricated structures in DPHPA produced by overlapping two of the 1st order beams with the central zero order region diffracted from the phase pattern in (a) displayed on the SLM. The red arrow indicates the pattern shifting direction. The dashed blue line indicates the location of lattices if there is no pattern shifting. Scale bar is for 8 μm.

When the square pixels are periodically arranged along the horizontal and vertical directions as in the checkerboard pattern in Figure 1a, the interference pattern is a square lattice that is aligned at 45 degrees relative to the horizontal direction as shown in Figure 1d. However the fabricated defect line in the PhC is still in the original orientation as in the designed phase pattern. In order to register the defect lines along the lattice vector directions (orientated 45 degrees relative to horizontal or vertical direction) in the PhC lattice, the orientation of the line defect in the phase pattern is rotated 45 degrees from the horizontal (or vertical) axis as shown in Figure 5a. The ordinarily black pixels along a diagonal line in the checkerboard phase pattern are changed into white pixels, creating the line defect phase. Figure 5b shows an enlarged view of a portion of the line defect in the background phase pattern. The red and blue squares indicate the unit cells from which the phase shift is calculated. In the unit cell of solid red squares, the diffracted beam 3 carries the information of the shifted phase relative to the background phase pattern. Diffracted beams (1 and 2) carry the shifted phase information for the unit cells in dashed blue squares and in solid blue squares, respectively. The first order diffracted beams (1 and 2) are blocked by the Fourier filter at the *f*_1_ Fourier plane, so only the unit cells indicated by the red squares in Figure 5b contribute to the shifting of the lattice due to the presence of the defects. The red arrows indicate the direction of the lattice shift in the interference pattern. Figure 5c shows an optical microscope image of the fabricated PhC lattices with line defects in DPHPA. In the image, only photonic lattices on one side of line defects are shifted away from the defect as indicated by the red arrows in Figure 5c and the lattices on the opposite side are not distorted, in very good agreement with the prediction in Figure 5b. By orienting the defect at 45 degrees and using 2 + 1 interference, several improvements have been realized including (a) alignment of the defect with the lattice side direction (instead of diagonal direction) of the background lattice square and (b) the shifting is only in one side of the defects. 

## 6. Registering the Functional Line Defect in Square PhC Lattice

**Figure 6:**
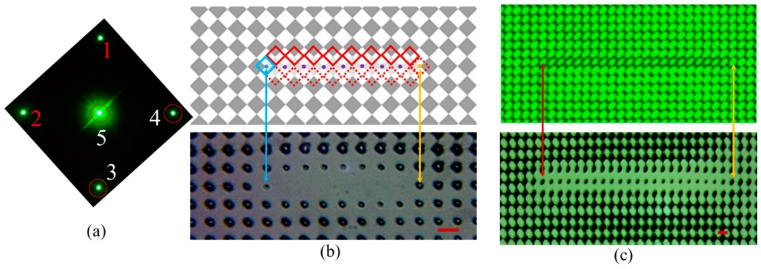
(**a**) CCD image of the diffraction pattern in the Fourier plane *f*_1_. All *images* are rotated 45 degrees to orient the defect along the horizontal direction. Beams (1 and 2) were filtered out with a mask. (**b**) The phase pattern displayed on the SLM and an optical microscope image of the fabricated structure. The background of the SLM image consists of single pixels of grey levels 254 and 158 and in the defect, the grey pixels have been changed to 224. (**c**) CCD image of the interference pattern and optical microscope image of the recorded interference generated by the phase pattern in (b), however, with a longer defect. Scale bars are for 5.65 μm as measured by atomic force microscope.

After fully understanding the lattice shift due to the “defect-content” and “shifted-phase” in the diffracted beams, we improved the design from Figure 5a. The new design includes a defect with a smaller relative phase shift and utilizes the difference in diffraction efficiency between the defect region and the background. Three beam (3, 4 and 5 as shown in Figure 6a) interference was used. Gray levels of 158 and 254 were used for the dark pixels and white pixels in the background phase pattern, respectively. These two numbers are selected due to the high diffraction efficiency of 12.3% of the first order diffracted beams relative to the total power reflected by the SLM. The dark pixels along the defect line in Figure 6b-top were changed to a light gray with a gray level of 224. A square phase pattern with gray levels of 224 and 254 has a low diffraction efficiency of just 1.6% for the first order diffracted beams. The dashed squares near the defect line indicate the unit cells that do not carry the shifted phase information in the diffracted beams 3 and 4, relative to beams diffracted from the background phase pattern. The solid red squares are the unit cells that carry the shifted-phase information and will shift the lattice vertically up and the blue solid square is for the unit cell that shifts the lattice to the left. However the phase shift of 2π × Δ = ((228−158)/255) × 0.25 × 2π is three times smaller than the phase shift of ((255–30)/255) × 0.25 × 2π in Figure 5b. Figure 6b-bottom shows the fabricated defect line in the background PhC lattice. Due to the small phase shift, the PhC lattices near the defect line are almost registered with the background lattice. In the middle of defect line, we can see void lattices due to the low diffraction efficiency. For the lattice at the ends of the defect line indicated by the blue and yellow arrows, the unit cells have half (158, 254) and half (224, 254) pixels. These two lattices survive after the holographic lithography. The lattice indicated by the blue arrow is smaller (or weaker) than the lattice indicated by the yellow arrow due to the pattern shifting in the former lattice. Overall, halls at the boundaries of the line defect are smaller than the other due to the diffraction efficiency. This effect can be removed if the phase pattern at the boundaries is designed to shift the pattern from the background toward the boundaries to compensate the low diffraction efficiency. Under an exposure with a longer exposure time, the lattice in the middle of the defect line can also survive. Figure 6c-top shows a CCD image of the interference pattern with a defect line. Figure 6c-bottom shows a fabricated defects-integrated PhC lattice in DPHPA showing a smaller (or weaker) but visible defect lattices. These results clearly demonstrate that a desired and functional defect lattice can be registered into the background lattice through direct imaging. The similar method can be used to make more complex defect structures in 2D such as ring resonators and resonant add-drop filters or couplers [6,7] or even to 3D if an objective lens and a thick photoreist film are used.

## 7. Conclusions

The direct imaging of designed defects in PhC lattices has been achieved by utilizing the high resolution capability of the SLM. We understand the defect related pattern shifting near the designed defect through the theoretic prediction and experimental validation. By reducing the relative phase shift and incorporating the different diffraction efficiency for the defect line and background phase pattern, we have simultaneously fabricated the designed defects in PhC lattice where the defect lattices are registered with the PhC lattice. These studies mark a step toward a fast, single step approach for holographic fabrication of photonic circuits embedded in PhCs.
